# Long-term exposure to nanoplastics reduces life-time in *Daphnia magna*

**DOI:** 10.1038/s41598-020-63028-1

**Published:** 2020-04-06

**Authors:** Egle Kelpsiene, Oscar Torstensson, Mikael T. Ekvall, Lars-Anders Hansson, Tommy Cedervall

**Affiliations:** 1 Department of Biochemistry and Structural Biology, Lund University, Lund University, P.O. Box 118, SE-221 00 Lund, Sweden; 2 Department of Biology/Aquatic ecology, Lund University, SE-223 62 Lund, Sweden; 3 NanoLund, Lund University, Box 118, SE-221 00 Lund, Sweden

**Keywords:** Ecology, Environmental sciences

## Abstract

Plastics are widely used in todays society leading to an accelerating amount of plastic waste entering natural ecosystems. Over time these waste products degrade to micro- and, eventually, nanoplastic particles. Therefore, the break-down of plastics may become a critical threat to aquatic ecosystems and several short term studies have demonstrated acute toxicity of nanoplastics on aquatic organisms. However, our knowledge about effects of chronic or life-time exposure on freshwater invertebrates remains elusive. Here, we demonstrate results from life-time exposure (103 days) of a common freshwater invertebrate, *Daphnia magna*, exposed to sub-lethal concentrations of polystyrene nanoparticles. 53 nm positively charged aminated polystyrene particles were lethal at concentration of 0.32 mg/L which is two magnitudes lower than previously used concentrations in short-term (24 h) tests. At this concentration the life-time of individuals was shortened almost three times. Negatively charged carboxylated 26 and 62 nm polystyrene particles, previously demonstrated to be non-toxic at 25 and 50 mg/L concentrations in short-term tests, were toxic to *D. magna* at all concentrations used in our long-term study. Although total reproductive output was not significantly affected at increasing concentrations of polystyrene nanoparticles, there was a decreasing trend in the number of offspring over their life-time. Hence, in order to understand how the potential future environmental problem of nanoplastic particles may affect biota, long-term or life-time studies resembling environmental concentrations should be performed in order to provide information for predictions of future scenarios in natural aquatic environments.

## Introduction

Plastic materials are of remarkable benefit for modern society due to their low price, easy manufacturing and practical function in a multitude of daily used products^1^. At a global scale, plastic production has increased tremendously over the past years^2^, and will likely continue increasing. Lebreton *et al*.^3^ estimated that between 1.15 and 2.41 million tonnes of plastic waste enter oceans every year. Plastics can degrade into a wide range of sizes, including micro- (<5 mm) and nano-sized (<100 nm) particles. Biological degradation^4^, exposure to ultraviolet radiation, and abrasion^5^ are processes taking place under natural conditions. Nanoplastics in nature has in one case been reported^6^ and the presence of styrene oligomers^7,8^ indicates continuing degradation of polystyrene in nature. Furthermore, ordinary polystyrene products submitted to mechanical forces^9^ or ultraviolet radiation^10^ in laboratory conditions release nanosized particles. Pollution by micro- and nanoplastics constitutes a potential threat to aquatic ecosystems^11,12^. Due to their small size, plastic particles might be ingested by organisms at the lower end of the food chain and can be thansferred by feeding to top consumers^13,14^. Several studies have shown that plastic particles of various sizes can be ingested by aquatic organisms causing tissue damage^15^ or even death^16^. Therefore, plastic pollution in aquatic environments and its potential impact on aquatic life has recently been recognized as an issue of considerable concern for society, as well as for ecosystem functioning^16^.

Although many studies addressing microplastic pollution have focused on marine environments^17,18^, recent reports have shown that microplastics can also be found in freshwater ecosystems^19,20^. Previous studies have reported microplastic ingestion by freshwater invertebrates such as tubificid worms^21^ and amphipod crustaceans^22^. Previously it has been observed that ingestion of nanoplastic particles may disturb fish feeding behavior and alter their metabolism^23–25^, as well as induce oxidative stress and tissue damage^26,27^.

Several ecotoxicological studies have used the freshwater crustacean *Daphnia magna* as study organism^28–31^. *D. magna* is a filter feeder and plays a key role in freshwater food chains as a food source for many aquatic organisms^32^. Previous studies have shown that *D. magna* can ingest nano- and microplastic particles ranging in size from 20 nm to 5μm^28,30^ and that *D. magna* show reduced reproduction after 21 days of exposure to 70 nm polystyrene particles^33^. Exposure to microplastics can also alter feeding behavior^31^, reduce growth rate or lead to immobilization in *D. magna*^34^.

The small size of plastic particles appears to be an important factor behind toxicity^24,29^. Mattsson *et al*.^24^ showed that survival of *D. magna* was significantly affected after acute exposure to concentrations ranging from 75 to 150 mg/L of 52 nm aminated polystyrene nanoparticles. Similarly, after exposure to 100 mg/L of positively and negatively charged polystyrene particles, *D. magna* neonates were all immobilized after 24 h exposure^29^. Additionally, exposure to 70 nm polystyrene particles negatively affected reproduction and body size of *D. magna* at concentrations of 0.22 and 103 mg/L^33^, and 52 nm polystyrene nanoparticles, at a concentration of 5 mg/L, reduced hatching rate and caused abnormal embryo development in *D. galeata*^35^.

Despite several acute toxicity tests showing negative effects on freshwater and marine zooplankton^28,34,36,37^, surprisingly little is known about the long-term biological and ecological effects of nanoplastics. Therefore, the aim of our study was to address the potential effects on life history traits (survival and reproduction) in *D. magna* to life-time exposure to three smallest commercially available polystyrene particles sizes.

## Results and Discussion

**Figure 1:**
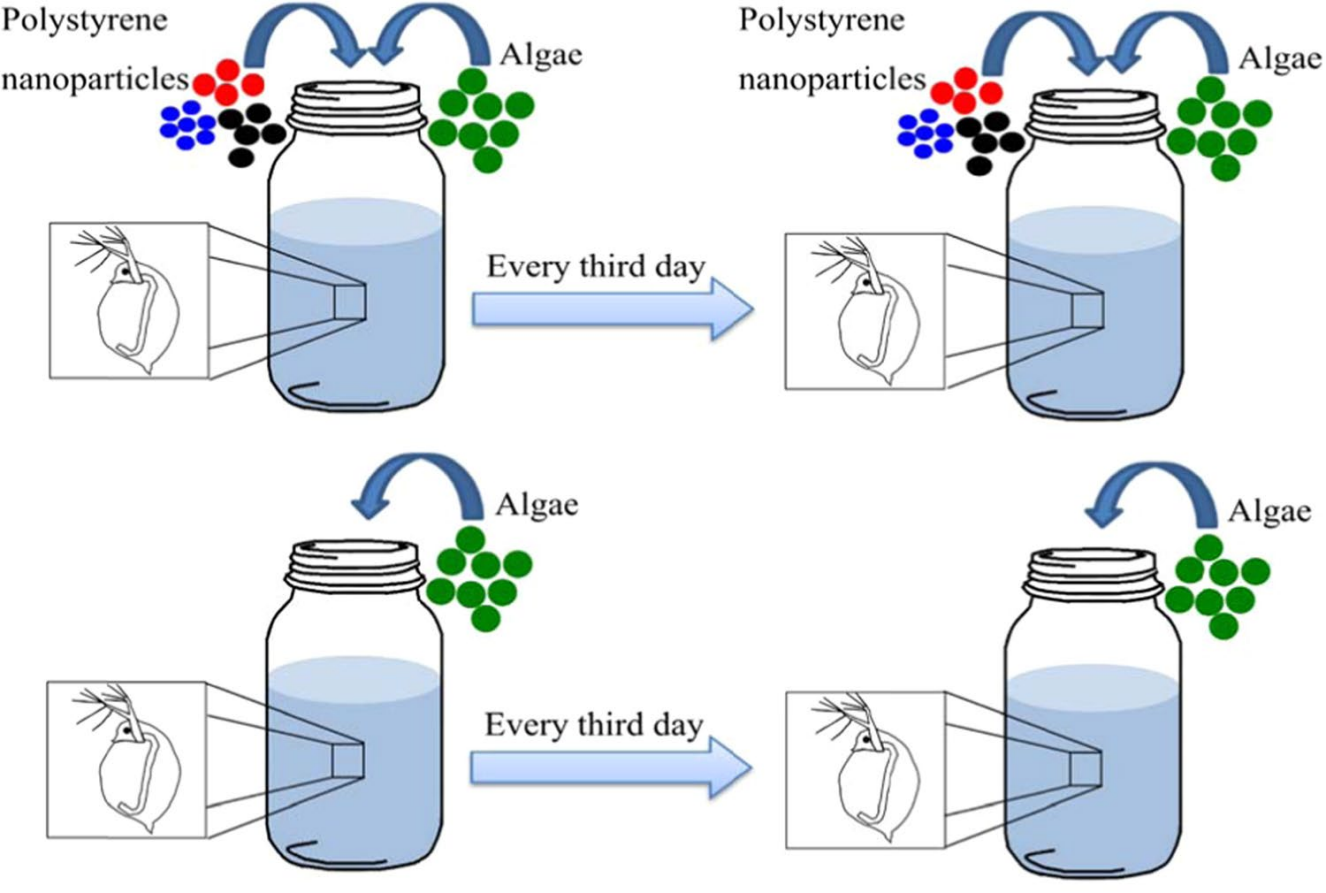
Schematic representation of long-term toxicity test. In total, there were ten replicates in each treatment. During the exposure to polystyrene nanoparticles, alive *Daphnia magna* individuals were transferred every third day to 100 mL glass beakers with 80 mL total volume of fresh medium, containing 2.5 mL of food (algae), with (treatment) or without (control) particles. Nanoparticles were dialyzed prior the experiments and particle sizes were measured during exposure using DLS. Algae concentration and water pH values were measured every time *D. magna* was transferred.

It has previously been shown in acute 24 h tests that small (50 to 60 nm) positively charged aminated polystyrene nanoparticles (PS-NH_2_) are the most toxic particles among the polystyrene nanoparticles tested on *D. magna*^24^. Therefore, 53 nm PS-NH_2_ nanoparticles were chosen in the present study to determine the lowest concentrations of nanoparticles observed causing mortality of *D. magna* in life-time exposure. Two to five day-old *D. magna* were isolated and exposed to polystyrene nanoparticles (Fig. 1) throughout their entire life-time, which for the oldest animal was 103 days. A concentration of 0.32 mg/L was chosen based from preliminary studies with aminated polystyrene nanoparticles (data not shown). In order to compare differently charged nanoparticles of specific surface areas, we increased concentrations for 62 and 26 nm carboxylic modified particles.

**Figure 2:**
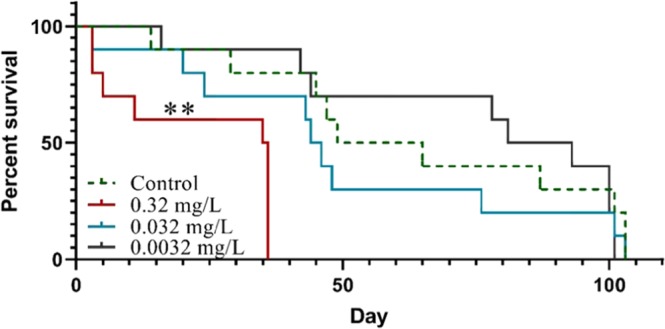
Survival of *Daphnia magna* exposed to different concentrations of 53 nm PS-NH_2_ throughout their life-time. Asterisk indicates significant difference compared to the control group estimated over the whole study period, **p < 0.01.

*D. magna* individuals exposed to 0.32 mg/L of 53 nm PS-NH_2_ showed an increased mortality (χ^2^_(1)_ = 10.19, *p* < 0.01) compared to the control group, while lower concentrations (0.032 and 0.0032 mg/L, Fig. 2) did not have any significant effects (χ^2^_(1)_ = 0.89 and 0.089, respectively, *p* > 0.05, Fig. 2). The lowest lethal concentration in the present study (0.32 mg/L) was 78 times lower compared to the lowest lethal concentration (25 mg/L) previously used in acute tests^24^.

**Figure 3:**
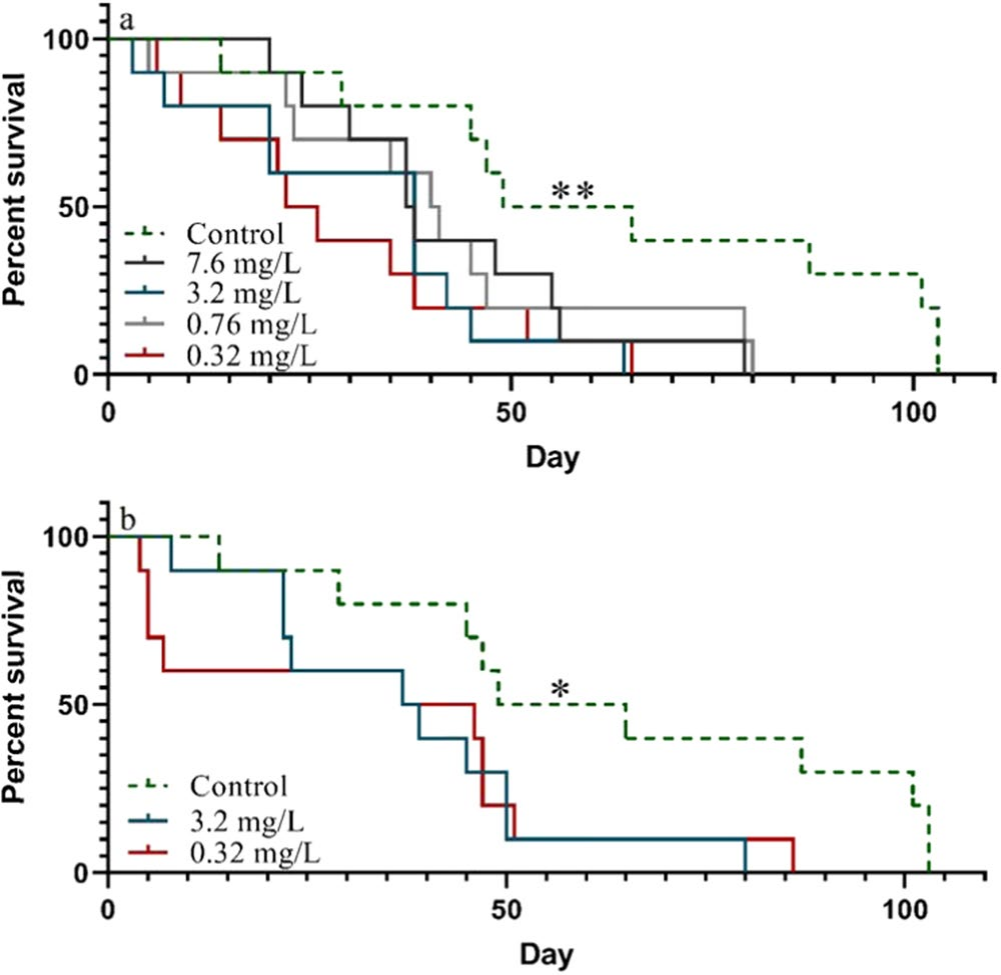
Survival of *Daphnia magna* exposed to 62 nm PS-COOH (**a**) and 26 nm PS-COOH (**b**) particles throughout their life-time. Asterisks indicate significant differences throughout the study period compared to the control group, *p < 0.05, **p < 0.01. Asterisks added on the control group indicate that all treatments were significantly different from the control group.

We also addressed the question if polystyrene nanoparticles that did not induce mortality in acute test^24^ is toxic at long-term (life-time) exposure. Negatively charged carboxylated polystyrene nanoparticles (PS-COOH) at the sizes 26 and 62 nm have been shown to be non-toxic in 24 h acute tests at concentrations up to 400 mg/L^24^. However, after long term exposure to lower concentrations (7.6, 3.2, 0.76 and 0.32 mg/L) of 62 nm PS-COOH in our study, *D. magna* showed a significant decrease in survival (χ^2^_(1)_ = 3.85, 8.03, 4.55 and 6.89, respectively, *p* < 0.05, Fig. 3a). Similarly, *D. magna* showed a significantly reduced survival rate than in the control when exposed to both 3.2 and 0.32 mg/L of 26 nm PS-COOH (χ^2^_(1)_ = 4.51 and 5.04, respectively, *p* < 0.05, Fig. 3b). For none of the sizes sub-lethal concentrations were reached and we may therefore conclude that although these carboxylated polystyrene particles were not considered toxic at short-term 24 h exposure^24^, they are indeed lethal at similar concentrations as the aminated particles at prolonged exposure.

Interestingly, there was an apparent reversed concentration dependency in survival between the lowest (0.32 mg/L) and highest (7.6 mg/L) concentrations of 62 nm PS-COOH. It could be speculated, especially as the polystyrene nanoparticles are mixed with the algae, that the exposure scenario was influenced by differences in nanoparticle concentrations, e.g. aggregation and/or faster sedimentation. Sedimentation was shown to be an important factor affecting exposure scenarios in a life-time test evaluating the effects of tungsten carbide nanoparticles^38^. However, in the present study, no sedimentation was observed over 48 h at a particle concentration of 7.6 mg/L mixed together with algae (Fig. S1). Furthermore, no particle aggregation, measured by dynamic light scattering (DLS), was observed in neither the lowest, nor the highest concentrations (Table S1). Another possible difference in exposure scenario is that the binding of organic molecules to the particle surfaces changes the toxicity of the particles. This effect has been shown for polystyrene particles pre-incubated in algae and in media containing molecules secreted from *D. magna*^29,33^. Increasing the particle concentration from 0.32 to 7.6 mg/L causes an increase in added particle surface area from 2.4 × 10^11^ to 56.4 × 10^11^ µm^2^ which may affect which type and how much organic material is bound to the particles.

**Figure 4:**
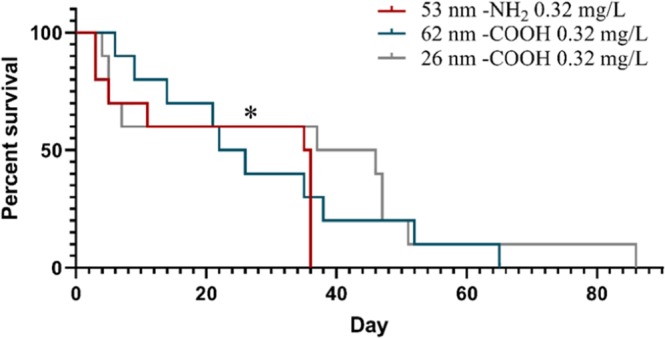
Comparison of *Daphnia magna* survival during life-time exposure to 53 nm PS-NH_2_, 62 nm PS-COOH and 26 nm PS-COOH at 0.32 mg/L. Asterisk indicates significant difference compared to the control group throughout the study period, *p < 0.05.

A comparison between the effect of 53 nm PS-NH_2_, 62 nm PS-COOH and 26 nm PS-COOH at 0.32 mg/L, revealed a significant difference in the survival of *D. magna* between 53 nm -NH_2_ and 26 nm -COOH treatments (χ^2^_(1)_ = 3.88, *p* < 0.05, Fig. 4). This implies that although the PS-COOH was shown to be toxic in the life-time experiments, but not in acute tests^24^, there is still a charge dependent toxicity. Generally, the positively charged PS-NH_2_ have been shown to be more toxic to *D. magna*, which might be due to a stronger interaction with the negatively charged *Daphnia* cell membrane^29^. It has also been shown that 50 nm PS-NH_2_ particle induces apoptosis in a variety of cells, while negatively charged nanoplastic particles did not have a significant effect^39^.

The accumulation of polystyrene nanoparticles in the body of *D. magna* has previously been demonstrated using fluorescent nanoparticles^29^, including the uptake of 20 and 70 nm particles^25,40^, as well as the accumulated body burden after 21 days exposure to 100 nm fluorescent polystyrene particles^37^. However, no data is available for the accumulation of non-fluorescent polystyrene nanoparticles. In order to document any microscopic changes during the life-time exposure, microscopic images were taken after death of several randomly chosen *D. magna* individuals (n = 3 for each treatment) that died after 30 to 100 days of exposure to different concentrations and sizes of PS-NH_2_ and PS-COOH. In some of the *D. magna* exposed to 53 nm PS-NH_2_ and 62 nm PS-COOH the gut contents were blackish (Fig. S2-B-C), which was not seen in individuals exposed to 26 nm PS-COOH (Fig. S2. D). These observations might suggest an accumulation of nanoplastic particles in some of the exposed organisms. This was not observed in any of the photographed individuals from the control group, where the guts instead had greenish contents from algal feeding (Fig. S2. A). Accumulation of nanoplastics in the gut of several organisms has previously been observed. For example, Torre *et al*.^41^ noted that after 48 h exposure negatively charged particles were accumulated in the digestive tract of sea urchin embryos, whereas positively charged nanoplastic particles were more dispersed in the gut. Nanoplastic particles aggregates have also been observed in *D. galeata* exposed to 52 nm polystyrene nanoparticles^35^. Similarly, Jemec *et al*.^28^ showed that polyethylene terephthalate textile microfibers were present in the gut of tested *D. magna* after 48 h exposure. Microplastic particles were also seen in the gut of exposed *D. magna* after 24 h test to particle concentrations of 12.5–400 mg/L, while the guts of control animals were greenish^34^.

In our study, the total number of offspring produced during the whole exposure time in treatments and the control group were not significantly different, neither by nanoparticle size nor concentration used within the same time period (*p* > 0.05 one-way ANOVA, Table S2). Similary, Rist *et al*.^37^ showed that reproduction was not effected after 21-day exposure to micro- and nanoplastic particles. However, at increasing concentrations of polystyrene nanoparticles, there was a decreasing trend in the number of offspring over their life-time (Table S3). Similarly, Besseling *et al*.^33^ also observed that increasing concentrations reduced the number of *D. magna* offspring. *D. magna* exposed to 0.1 mg/L of 1–5 μm microplastics of polymer microspheres for 21-day showed a significant reduction in reproduction^42^. Rist *et al*.^37^ showed that there was no difference in time to first offspring when *D. magna* were exposed to micro- and nanoplastic particles for 21 days, whereas Pacheco *et al*.^43^ observed a delay in the first brood release in *D. magna* after exposure to 1–5 μm microplastics. Likewise, Ekvall *et al*.^38^ showed a significant delay in time to first brood in *D. magna* exposed to tungsten carbide nanoparticles.

The majority of the published studies focus on acute, short-term, tests at high plastic particle concentrations^24,29,34^, whereas long-term toxicity studies on nanoplastics are rare, despite long-term, even life-time, exposure to low concentrations is the rule as nanoparticles enter natural ecosystems. Therefore, our understanding on how life-time exposure to nanoplastic particles affect organisms in aquatic food chains still remains elusive. Potential effects on aquatic organisms, such as zooplankton, may have considerable consequences for the function of aquatic food webs in which these organisms play a key role. In natural environments aquatic organisms are exposed to different sizes of plastic particles during their whole life-span. Our life-time experimental set-up does not only demonstrate toxicity of nanoplastic particles at relatively low concentrations, but also reveals toxicity of nanoplastics that are apparently non-toxic in standardized 24 or 48 h acute tests even at very high concentrations. Furthermore, in many cases mortality occurs after the standardized long-term 21-day tests. This clearly suggests that routine, standard test times may not be enough to assess the severity of plastic particles in our environment. Hence, by introducing life-time exposure tests we were here able to identify lethal effects at concentrations almost two magnitudes lower than previously shown^29^. Moreover, mortality may not be optimal to assess the lowest concentration of nanoplastic particles that will negatively affect the environment. Slow uptake of nanoplastics at low concentrations allow for accumulation of particles by the individuals, whereas high concentrations of nanoplastics in acute tests may rip off tissue or deplete the digestive system of neseccary enzymes^44–46^. In the future there is a need for mechanistic studies of the long-term toxicity in order to be able to properly assess the environmental risk, as well as the risk of different kinds of plastic particles.

Although the relevant concentrations of nanoplastic particles have, due to methodological constraints, not been determined, we here use relatively low concentrations of nanoplastic. We conclude that long-term exposure to low concentrations of nanoplastics material may provide considerably different outcomes with respect to toxicity than short-term, acute tests at high concentrations. Since long-term, or even life-time exposures may even already be ongoing in many regions of the world, our results have considerable implications for our use and manufacturing of plastic materials.

## Material and Methods

### Study organisms

The *Daphnia magna* culture used originates from Lake Bysjön, southern Sweden (55°40′31.3″N 13°32′41.9″E), and has been kept in the laboratory for several hundred generations. The culture was fed three times per week with an algae diet mainly composed of the green algae *Scenedesmus* sp. The algal culture was filtered once a week through 20 μm mesh filter to remove larger algal species, such as cyanobacteria, from the culture and fed with 250 µL of liquid plant nutrient, of which 100 mL contains 5.1 g nitrogen, 1.0 g phosphorus and microelements. All cultures were maintained at 18 °C at a 8:16 h light/dark photoperiod.

### Nanoparticles preparation and characterization

The smallest commercially available positively (aminated, diameter size of 53 nm) and negatively charged (carboxylated, diameter sizes of 26 and 62 nm) polystyrene particles were purchased from Bangs Laboratories Inc. (www.bangslabs.com). Prior to the start of the experiments, particles were diluted to 10 mg/mL and dialyzed in Standard RC Tubing, Dialysis Membrane (MWCO: 3.5 kD) for 24 h in 10 L of MilliQ water. Water was changed at least 4 times during the dialysis, which was performed to separate the nanoparticles from the solvent creating a stock solution suitable for toxicity testing of nanoparticles. The particle sizes were measured in triplicates using DLS on DynaPro Plate Reader II (Wyatt instruments, USA) directly after dialysis and every third day during experiments to ensure that particle sizes did not change during the study. No changes in particles sizes were observed during the experiment (Table S4.1-3). To quantify particle sedimentation rate, absorbance of particle suspension mixed with algae cells was measured. 1 mL of the medium solution was added to a quartz cuvette and the absorbance was measured at 200–250 nm by a flash light through a fixed point, 0.8 mm in diameter, during 48 h using a ProbeDrum spectrophotometer (Probation Labs, Lund, Sweden). We recorded no change in absorbance, suggesting that sedimentation did not occur (Fig. S1).

### Exposure to nanoparticles

A life-long experiment (the median life-time for a control group was 64.3 ± 32.5 days) on *D. magna* was performed to analyze effects on life history traits (survival and reproduction) when exposed to different concentrations of polystyrene particles of three different sizes (Table 1). Different concentrations were chosen to determine the lowest concentration of polystyrene nanoparticles causing mortality in *D. magna*. Two-five days old *D. magna* individuals from the same population were isolated and randomly assigned to the different groups. Gender determination was not possible since handling and microscopic examination induced high mortality at this early age. Each individual was put in a 100 mL uncovered glass beaker with 80 mL total volume (n = 10 for each treatment), filled with tap water which had been aerated for 24 h prior to the start of the experiment in order to increase the oxygen level. Aeration was repeated prior to the medium exchange. During the exposure to nanoplastic particles, alive *D. magna* individuals were gently transferred to fresh medium by using a 1 mL plastic pipette with a removed tip to reduce handling stress, every third day. The fresh medium contained 2.5 mL of food (algae), with (treatment) or without (control) nanoparticles (Fig. 1). Water with nanoplastic particles was mixed thoroughly each time before adding it to exposure jars to ensure that particle number did not vary between samples. Experimental cultures were maintained at 18 °C at a 8:16 h light/dark photoperiod.

**Table 1:** Characteristics for particles used.

Surface modification	Diameter size (nm)	Concentration (mg/L)	Specific surface area (μm^2^/mg)	Particles/mL (at concentration of 0.32 mg/L)	Surface charge
Aminated (-NH_2_)	53	0.32, 0.032, 0.0032	1.11 × 10^11^	3.91 × 10^9^	Positive
Carboxylated (-COOH)	26	3.2, 0.32	2.21 × 10^11^	3.31 × 10^10^	Negative
	62	7.6, 3.2, 0.76, 0.32	9.29 × 10^10^	2.44× 10^9^	

Algae stock culture was filtered through 20 μm mesh filter, diluted with tap water to keep algae concentration stable throughout the experiment (Table S5). The concentration of algae (chlorophyll *a*) in the stock culture was assessed in triplicates prior to transferring *D. magna* to fresh medium using AlgaeLabAnalyser (bbe Moldaenke, GmbH). 2.5 mL of this algae culture was then added to *D. magna* individuals each time individuals were transferred. The tap water was aerated 24 h prior individuals transfer and used to make new particle containing media, and control group (fresh and 3 days old) samples. The pH remained stable in all samples during the experiment (Fig. S3). To document any morphological changes in the exposed animals, photos were taken using a stereo microscope (Olympus SZX7) of randomly chosen *D. magna* individuals that died after 30 to 100 days of exposure to different concentrations, charges and sizes of nanoparticles. At least three photos were taken for each treatment during the experiment. The survival rate of *D. magna* was checked daily, while reproduction rate was checked every third day. Offspring were counted and removed from glass beakers every third day.

### Statistical analysis

Kaplan Meier survival curves analysis were performed using statistical computing software GraphPad Prism version 8.0.0 (224) for Windows, GraphPad Software, Inc., www.graphpad.com, and one-way ANOVA was used to test for differences in reproduction output in R version 3.6.1, www.r-project.org.

## Supplementary information

Supplementary information.
