# Supplementary material for: Long-term exposure to nanoplastics reduces life-time in Daphnia magna
Source: Sci Rep. 2020 Apr 6;10:5979. doi: 10.1038/s41598-020-63028-1 (PMC7136239; doi:10.1038/s41598-020-63028-1)
Supplement: Supplementary file 1 — Supplementary information. [file 41598_2020_63028_MOESM1_ESM.pdf]

Supplementary Information

Long-term exposure to nanoplastics reduces life-time in *Daphnia magna*

Egle Kelpsiene[1,3*], Oscar Torstensson[1], Mikael T. Ekvall[2,3], Lars-Anders Hansson[2,3], and Tommy Cedervall[1,3]

[1]*Department of Biochemistry and Structural Biology, Lund University, Lund University, P.O. Box 118, SE-221 00 Lund, Sweden*

[2]*Department of Biology/Aquatic ecology, Lund University, SE-223 62 Lund, Sweden*

[3]*NanoLund, Lund University, Box 118, SE-221 00 Lund, Sweden*

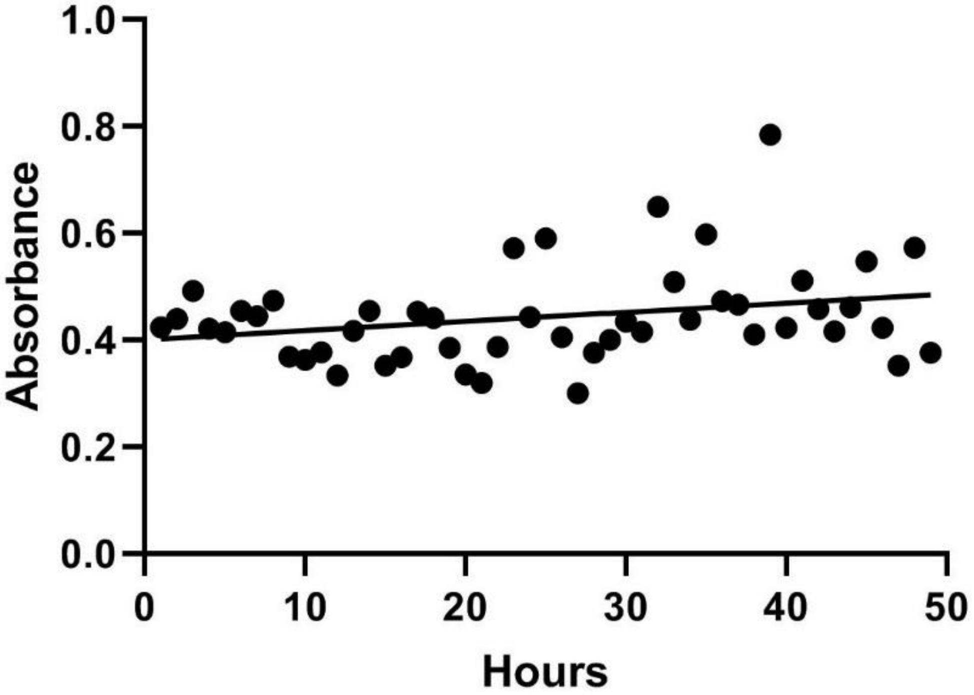

Fig. S1. Absorbance of 62 nm PS-COOH (7.6 mg/L) particle mixed together with algae over 48 h. Absorbance was measured at 200-250 nm as polystyrene has a strong absorbance maximum at 230 nm. No change in absorbance indicates that particle sedimentation did not occur. Sedimentation was measured only for the highest concentrations of 62 nm PS-COOH as an apparent reversed concentration dependency was noticed in survival of *Daphnia magna*. The sedimentation velocity for polystyrene nanoparticles would be more than 10 000 years, which is calculated according to an equation which includes depth of solution, viscosity of solution, radius of particle, density of particle, density of solution, the acceleration due to gravity.

Table S1. Radius and % polydispersity of 0.32 and 7.6 mg/L of 62 nm of PS-COOH after 48 h measured in triplicates by DLS. No particle aggregation was observed in measured concentrations. Aggregation was measured for the lowest and highest concentrations of 62 nm PS-COOH as an apparent reversed concentration dependency was noticed in survival.

Concentration (mg/L)	Radius (nm)	% DP
0.32	37.087	20.7
	48.751	7.1
	36.312	22.1
7.6	27.206	14.2
	28.368	12.3
	27.141	12.4

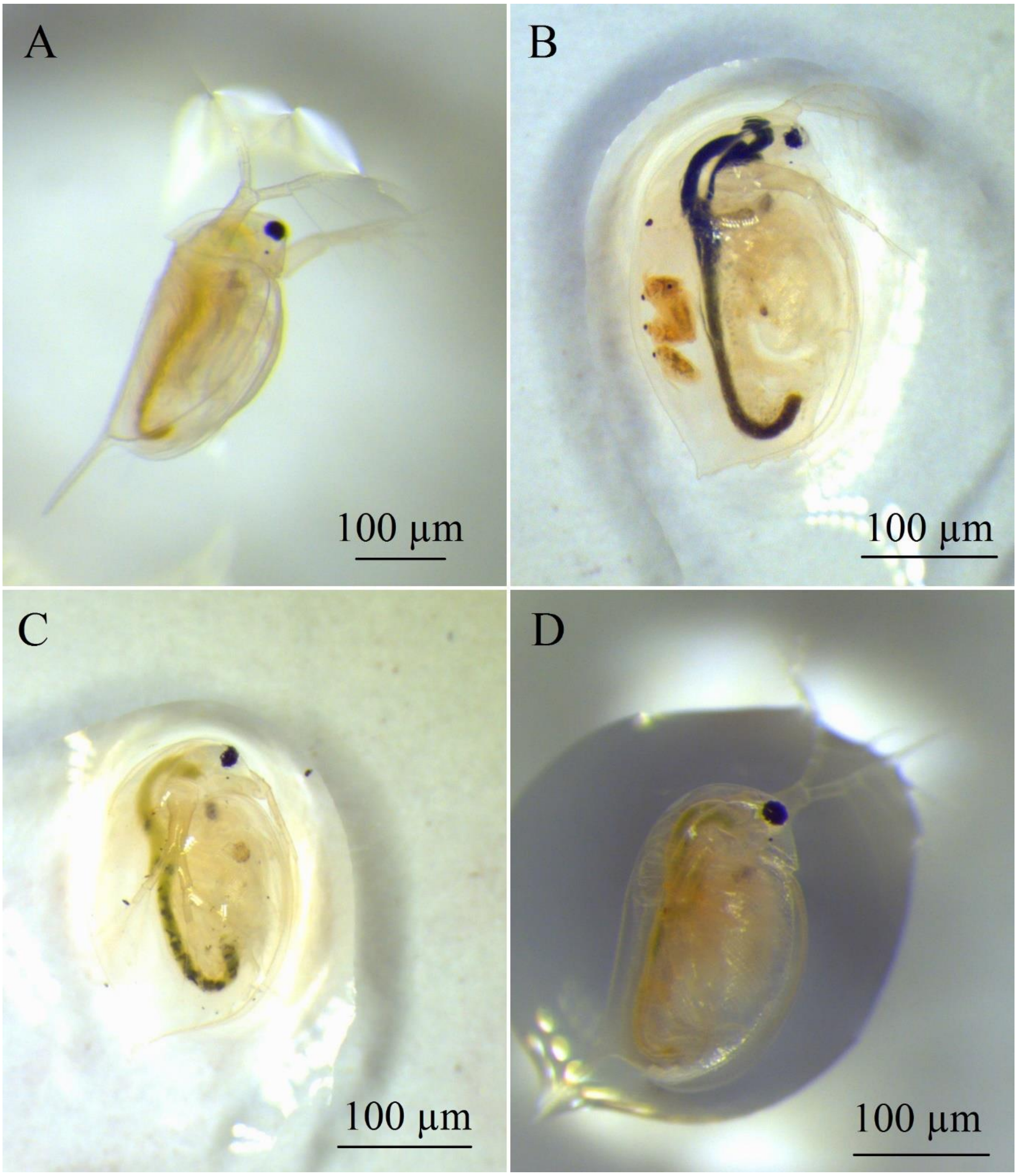

Fig. S2. *Daphnia magna* individuals in the control group (A) and after exposure to differently charged and differently sized polystyrene nanoparticles (53 nm PS-NH_2, B; 62 nm PS-COOH, C; 26 nm PS-COOH, D). Localized accumulation and blackish guts were seen in *D. magna* after exposure to 53 nm PS-NH_2 (B) and 62 nm PS-COOH (C). This was not seen in the control group. Pictures were taken for randomly chosen individuals after death, at least three

images were taken from each treatment. Individuals were photographed after 30 to 100 days of exposure.

Table S2. Mean number of offspring produced by *D. magna* replicates (N ≤ 10, as not all replicates were females) in the control group and exposed to 53 nm PS-NH_2, 62 nm PS-COOH, and 26 nm PS-COOH. The first brood was observed in all groups after 9 days from the beginning of the experiment. Empty vials indicate that there were no alive individuals left. Offspring were counted and removed from glass beakers every third day. Mean values were calculated together with standard deviation.

Day		9	12	15	18	21	24	27
Control		3.13±4.32	7.5±5.26	7±6.56	3.71±2.56	-	1±1.41	-
53 nm -NH_2	0.32 mg/L	3.6±3.78	8.25±5.91	6.75±6.8	3.75±2.5	0.5±1	0.25±0.5	2.25±1.71
	0.032 mg/L	2.22±3.42	6.67±5.98	8.22±5.4	3.44±2.92	0.63±1.19	1.57±1.9	-
	0.0032 mg/L	3.44±4.22	5.56±5.55	5.13±5.54	2.38±3.46	-	0.63±1.77	-
62 nm - COOH	7.6 mg/L	1.33±4	6.56±6.29	7.44±5.81	3±2.83	0.5±1.41	1.86±1.77	-
	3.2 mg/L	6.75±4.99	7.25±8.46	3.75±7.5	4.75±3.3	0.75±1.5	-	-
	0.76 mg/L	1±2.83	5.38±5.13	10±5.35	3±3.34	0.29±0.76	2±1.41	-
	0.32 mg/L	2.25±4.3	9.38±5.26	7.57±4.08	2.43±3.55	1.67±2.66	2±1.41	-

26 nm -COOH	3.2 mg/L	1.14±3.02	5±5.29	9.71±5.41	3.71±2.56	-	-	0.2±0.45
	0.32 mg/L	1.6±3.58	9.2±4.76	10.8±2.49	2±2.83	1.2±1.79	3.2±2.28	-
Day		30	33	36	39	42	45	48
Control		9.43±6.16	4.43±4.16	0.43±1.13	0.43±0.79	-	6±3.22	10.5±5.8
53 nm $-NH_2$	0.32 mg/L	4.5±5.26	8.25±6.99					
	0.032 mg/L	7.14±5.79	5.29±4.39	3.57±3.1	1.57±1.8	-	3±3.94	4.33±4.04
	0.0032 mg/L	7.38±4.63	5.38±4.96	1.75±3.41	0.38±0.52	-	0.57±1.51	2.14±3.93
62 nm -COOH	7.6 mg/L	8.67±5.05	1.5±2.35	3.6±3.58	0.4±0.89	-	-	-
	3.2 mg/L	11±1.63	3±3.83	0.75±1.5	1.8±2.49			
	0.76 mg/L	4.5±3.89	3.5±4.18	1.8±2.49				
	0.32 mg/L	7±1.41	2.75±5.5	4±3.16	1±1.41	-	-	-
26 nm -COOH	3.2 mg/L	8.6±4.98	4±3.94	2±2.83	3.75±0.5	1±2	6.67±4.73	8±9.54
	0.32 mg/L	7±5.87	5±4.24	1±2	1.75±2.06	1±2	6.5±1	7
Day		51	54	57	60	63	66	69

Control		-	15.25±4.03	8.75±4.11	-	1.5±1.73	-	2.67±2.52
53 nm -NH_2	0.32 mg/L							
	0.032 mg/L	0.33±0.58	15.67±8.08	4±3.61	-	1.33±1.53	-	1±1.73
	0.0032 mg/L	9.43±10.98	1.57±2.82	4±3.21	0.14±0.38	0.43±1.13	4±5.13	3.14±3.93
62 nm -COOH	7.6 mg/L	-	2±2.83					
	3.2 mg/L							
	0.76 mg/L							
	0.32 mg/L	-	-	-	1			
26 nm -COOH	3.2 mg/L	-	16	7	-	1	-	-
	0.32 mg/L							
Day		72	75	78	81	84	87	90
Control		-	2.67±4.62	1.33±2.31	-	-	3.33±2.89	1±1.41
53 nm -NH_2	0.32 mg/L							
	0.032 mg/L	-	1.5±2.12	3±1.41	-	-	-	2±2.83

	0.0032 mg/L	0.29±0.76	0.43±1.13	2.86±3.18	-	-	1.5±2.51	2.83±2.79
62 nm - COOH	7.6 mg/L							
	3.2 mg/L							
	0.76 mg/L							
	0.32 mg/L							
26 nm - COOH	3.2 mg/L	1	9	-				
	0.32 mg/L							
Day		93	96	99	102			
Control		-	-	3.5±4.95	-			
53 nm $-NH_2$	0.32 mg/L							
	0.032 mg/L	1±1.41						
	0.0032 mg/L	-	2±3.94	2±3.46				
	7.6 mg/L							

62 nm - COOH	3.2 mg/L							
	0.76 mg/L							
	0.32 mg/L							
26 nm - COOH	3.2 mg/L							
	0.32 mg/L							

Table S3. Reproduction efficiency in treated groups. There were no significant differences in reproduction efficiency between treated individuals compared to the control group within the same time period. Reproduction efficiency was calculated by dividing total number of offspring by number of females and survived days.

Treatment		Total number of offspring	Last survival day	Reproduction efficiency
53 nm $-NH_2$	0.0032 mg/L	505	99	0.63
	0.032 mg/L	453	99	0.51
	0.32 mg/L	156	33	1.18
62 nm -COOH	0.32 mg/L	241	45	0.67
	0.76 mg/L	256	39	0.72
	3.2 mg/L	156	39	1
	7.6 mg/L	267	57	0.52

26 nm -COOH	0.32 mg/L	248	48	1.03
	3.2 mg/L	308	75	0.59
Control		489	103	0.59

Table S4.1. 53 nm PS-NH_2 diameter size (nm) and % polydispersity measured in triplicates by DLS. Measurements were taken direct after particle dialysis (Day 0), and every third day during the exposure to ensure that particle size remained stable. Mean values were calculated together with standard deviation.

Day	0	3	6	9	12	15	18
Diameter (nm)	50.12±4.19	49.52±0.22	50.23±1.81	50.86±2.06	49.91±1.16	48.64±0.93	48.29±0.99
% DP	14.23±2.72	12.5±0.2	16.5±7.51	18.8±8.15	13.07±1.55	12.57±2.05	13.4±0.72
Day	21	24	27	30	33	36	39
Diameter (nm)	49.21±0.61	49.85±0.46	48.85±4.99	48.00±0.53	48.16±0.65	48.62±0.45	48.76±0.33
% DP	16.43±6.44	12.07±0.23	22.63±1.07	13.67±0.67	12.13±1.32	13.6±0.44	13.07±1.93
Day	42	45	48	51	54	57	60
Diameter (nm)	48.95±0.78	48.36±0.63	49.18±0.12	47.78±1.34	42.96±1.08	48.80±0.42	49.69±0.50
% DP	14.67±0.71	13.07±1.15	13.2±0.72	13.03±2.70	4.53±7.85	14.43±2.27	11.27±2.55
Day	63	66	69	72	75	78	81

Diameter (nm)	49.10±0.50	47.89±0.58	48.49±0.72	48.36±0.13	49.58±0.46	49.32±0.45	48.71±0.49
% DP	14.27±2.37	12.63±0.89	15.87±2.20	15.2±1.83	13.37±1.76	17.77±2.51	15.13±0.81
Day	84	87	90	93	96	99	102
Diameter (nm)	50.83±1.04	49.04±1.12	51.12±0.62	51.29±0.12	42.31±4.83	45.21±0.56	49.09±1.14
% DP	16.87±1.05	16.6±1.57	16.27±1.08	17.4±0.36	21.07±4.92	17.9±3.00	12.6±2.07

Table S4.2. 62 nm PS-COOH diameter size (nm) and % polydispersity measured in triplicates by DLS. Measurements were taken direct after particle dialysis (Day 0), and every third day during the exposure to ensure that particle size remained stable. Mean values were calculated together with standard deviation.

Day	0	3	6	9	12	15	18
Diameter (nm)	58.19±0.14	58.90±0.57	57.17±2.77	53.78±4.52	55.08±3.87	53.66±3.39	57.39±0.37
% DP	8.27±1.01	8.5±1.21	12.23±5.09	18±5.14	11.43±3.39	13.83±4.53	9.2±1.18
Day	21	24	27	30	33	36	39
Diameter (nm)	58.48±0.83	59.06±0.81	55.57±3.75	58.49±1.11	56.86±2.63	57.21±0.48	57.67±0.08
% DP	9.23±1.32	8.77±0.85	12.23±7.25	10.07±0.71	12.87±6.31	10.07±1.07	10.63±1.59

Day	42	45	48	51	54	57	60
Diameter (nm)	58.13±0.83	58.02±1.82	57.66±0.55	58.01±0.64	55.64±1.11	56.71±1.66	55.76±1.18
% DP	9.5±1.15	12.97±4.66	9.1±1.65	8.33±1.32	11.73±2.64	9.87±1.95	4.63±2.02
Day	63	66	69	72	75	78	
Diameter (nm)	57.01±1.36	50.62±0.18	57.66±0.72	57.99±1.73	57.21±0.41	58.05±0.37	
% DP	6.7±2.23	9.73±2.14	9.23±0.51	7.23±3.29	11.87±0.06	8.77±1.4	

Table S4.3. 26 nm PS-COOH diameter size (nm) and % polydispersity measured in triplicates by DLS. Measurements were taken direct after particle dialysis (Day 0), and every third day during the exposure to ensure that particle size remained stable. Mean values were calculated together with standard deviation.

Day	0	3	6	9	12	15	18
Diameter (nm)	26.05±1.65	25.43±0.31	25.44±0.29	25.95±0.23	25.59±0.45	25.26±0.06	25.75±0.18
% DP	14.53±1.99	16.87±1.95	16±2.42	17.87±1.63	16.27±2.11	15.47±1.25	15.43±1.01
Day	21	24	27	30	33	36	39
Diameter (nm)	25.39±0.39	25.4±0.61	25.12±0.56	24.9±0.06	25.39±0.23	24.76±0.24	25.18±0.33

% DP	17.13±2.15	16.5±1.47	14.37±2.15	13.37±0.47	15.2±0.61	14.1±0.17	16.13±3.93
Day	42	45	48	51	54	57	60
Diameter (nm)	25.15±0.53	25.29±0.49	24.98±0.42	25.56±0.06	25.61±0.61	24.79±0.57	25.54±0.39
% DP	15.53±3.72	14.97±1.89	13.97±0.75	16.2±2.51	16.07±4.38	13.1±5.3	14.9±2.55
Day	63	66	69	72	75	78	81
Diameter (nm)	25.51±0.24	25.31±0.28	25.14±0.18	25.19±0.15	24.99±0.06	25.4±0.12	24.92±0.23
% DP	15.93±1.05	15.47±1.72	14.37±1.59	13.57±1.08	14.53±1.00	15.93±4.13	13.7±0.36
Day	84	87					
Diameter (nm)	25.29±0.41	25.27±0.56					
% DP	14.73±0.78	16.23±0.96					

Table S5. Total algae and green algae concentrations used during the exposure to polystyrene nanoparticles. Data presented as mean values calculated together with standard deviation.

Day	0	3	6	9	12	15
Total algae conc. (μg/L)	542.17±14.26	531.45±10.25	545.01±8.20	530.57±0.93	544.98±0.41	549.06±3.32

Green algae conc. (μg/L)	65.38±10.20	59.07±5.68	65.30±5.56	150.42±11.25	438.21±0.65	344.42±2.39
Day	18	21	24	27	30	33
Total algae conc. (μg/L)	516.59±3.14	532.38±3.86	558.89±2.27	485.58±5.614	593.32±2.22	513.47±1.75
Green algae conc. (μg/L)	408.89±0.87	206.96±13.67	375.82±1.35	258.19±3.77	315.42±2.27	232.88±2.37
Day	36	39	42	45	48	51
Total algae conc. (μg/L)	519.39±1.36	490.15±4.51	550.17±6.28	549.13±9.56	545.01±4.08	429.51±0.73
Green algae conc. (μg/L)	302.63±1.56	184.01±15.55	71.76±4.01	68.80±5.45	70.28±2.69	95.41±9.30
Day	54	57	60	63	66	70
Total algae conc. (μg/L)	539.95±1.91	478.75±1.27	640.67±2.70	498.41±0.84	553.29±3.82	500.59±0.91
Green algae conc. (μg/L)	169.92±17.14	183.22±7.45	229.74±2.49	179.92±6.48	291.48±19.90	132.43±17.93
Day	73	76	79	81	84	87
Total algae conc. (μg/L)	567.35±0.82	345.56±8.57	459.2±0.84	446.77±0.67	521.05±1.69	595.65±2.91
Green algae conc. (μg/L)	240.95±8.56	330.92±6.75	287.02±5.46	134.27±9.58	358.92±9.99	96.55±1.38
Day	90	93	96	99		
Total algae conc. (μg/L)	657.29±1.75	568.2±1.67	566.94±5.31	375.19±1.11		

Green algae conc. (μg/L)	190.82±4.96	449.25±0.99	422.02±4.35	235.80±1.28		

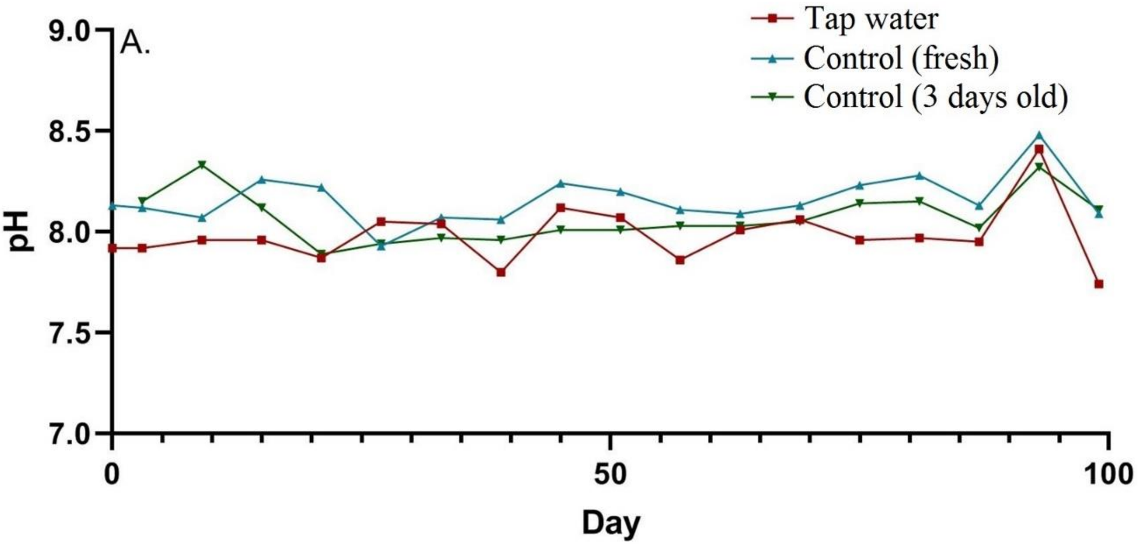

Fig. S3. pH during the exposure polystyrene nanoparticles. Tap water was aerated for 24 h prior to the start of the experiment and every time before the medium exchange.
